# CHANGES IN RURAL AND URBAN FAMILY CAREGIVING NETWORKS IN THE MIDWEST DURING THE COVID-19 PANDEMIC

**DOI:** 10.1093/geroni/igac059.777

**Published:** 2022-12-20

**Authors:** Sato Ashida, Hyunkeun Cho, Lena Thompson, Kristine Williams, Laura Koehly, Haley Schneider, Maria Donohoe, Lubna Hossain

**Affiliations:** University of Iowa College of Public Health, Iowa City, Iowa, United States; University of Iowa, Iowa City, Iowa, United States; University of Iowa, Iowa City, Iowa, United States; University of Kansas Medical Center, Lawrence, Kansas, United States; National Institutes of Health, Bethesda, Maryland, United States; University of Iowa College of Public Health, Iowa City, Iowa, United States; University of Iowa College of Public Health, Iowa City, Iowa, United States; University of Iowa College of Public Health, Iowa City, Iowa, United States

## Abstract

This study elucidates the changes in family caregiving networks during the COVID-19 pandemic and its implications on caregiver well-being. Eighty-two caregivers of individuals diagnosed with dementia within the past 2 years participated in this study to test a post-diagnosis intervention that provides a community care planning service that connects caregivers directly to community-based services. Caregivers completed telephone surveys at baseline and 3- and 6-month follow-up. The number of network members engaging in malfeasant (negative) social interactions increased by 0.798 every 3 months (p=0.002). Members engaging in uplifting interactions decreased, especially among intervention participants, by 1.93 every 3 months (p=0.047); urban caregivers reported greater decrease than rural (p=0.006). Participants in intervention group showed a trend for greater decrease in COVID-19 related distress (10-point scale) over time compared to control group (p=0.059); those with more members engaging in uplifting interactions reported lower distress (p=0.017) regardless of intervention status, network size, and rurality.

